# Study on the Utilization of Inpatient Services for Middle-Aged and Elderly Rural Females in Less Developed Regions of China

**DOI:** 10.3390/ijerph17020514

**Published:** 2020-01-14

**Authors:** Xiaotong Wen, Lanyue Cui, Fang Yuan, Xiaojun Liu, Mufeng Ouyang, Yuxiao Sun, Yuchen Liu, Yong Liu, Huiqiang Yu, Huilie Zheng, Yuanan Lu, Zhaokang Yuan

**Affiliations:** 1 School of Public Health, Nanchang University, Nanchang, Jiangxi Province Key Laboratory of Preventive Medicine, Nanchang 330006, China; 406530517824@email.ncu.edu.cn (X.W.); 13007231568@163.com (Y.L.); yuhuiqiang@ncu.edu.cn (H.Y.); zhenghuilie@ncu.edu.cn (H.Z.); yuanan@hawaii.edu (Y.L.); 2 Queen Mary School, Nanchang University, Nanchang 330006, China; cuilanyueliang@163.com; 3 Office of Public Health Studies, University of Hawaii at Mānoa, Honolulu, HI 96822, USA; fangy@hawaii.edu (F.Y.); oymufeng@gmail.com (M.O.); conicalverb@hotmail.com (Y.S.); renata2001bella@hotmail.com (Y.L.); 4 Global Health Institute, Wuhan University, Wuhan 430071, China; lxiaojun@aliyun.com

**Keywords:** less developed regions, rural, middle-aged and elderly, female, inpatient

## Abstract

The aim of this study is to understand the utilization of inpatient services and its contributing factors among middle-aged and elderly females (MAEF) in less developed rural regions. Five surveys were conducted between 2006 and 2014 with rural residents of Jiangxi by stratified cluster random sampling. Participant females included only those who were aged 45 and above. Complex sampling logistics analysis was performed to analyze the effect of three factors on inpatient service. Complex sampling logistics regression analysis revealed that the probability of hospitalization for the divorced or widowed females was significantly lower than that of married ones (aOR = 0.177, *p* < 0.05). However, the probability of early discharge was significantly higher among divorced or widowed females than married ones (aOR = 3.237, *p* < 0.05). In addition, females with chronic diseases were more likely to be hospitalized (aOR = 3.682, *p* < 0.05). Also, early discharge (aOR = 7.689, *p* < 0.05) occurred among the participants who should be hospitalized but were not hospitalized occurred (aOR = 3.258, *p* < 0.05). The continuous improvement of the new rural cooperative medical policy has promoted the utilization of inpatient services for the MAEF. Findings from this study emphasize the need to strengthen the prevention and treatment of chronic diseases among middle-aged and elderly women.

## 1. Introduction

There are still quite a few economically underdeveloped regions in China, which include 5 provinces in northwest China, 4 provinces in the southwest, and 8 in the central region. The population of those regions accounts for 58.19% of China’s total population, of which the rural population accounts for 41.43% [1]. There is a certain economical gap between these less developed regions and the eastern coastal areas, with low per capita income and poor infrastructure construction. Thus, the less developed region is the main focus in this phase. How to improve the health level of rural residents in less developed areas and achieve equalization of medical and health services have been a major concern [2,3,4].

In rural areas of economically underdeveloped areas, the industrial structure is dominated by agriculture, labor productivity is low, per capita income is low, and a large number of people flow to cities and developed areas for employment. Young people leave rural areas for better employment opportunities. A larger percentage of middle-aged and elderly females live in rural areas. Due to their older age and lower education level, middle-aged and older females find it difficult to find jobs in cities and developed areas, and thus, most of them stay in rural areas. More and more middle-aged and elderly females (MAEF) appear to lack the care of their families and become a vulnerable group that needs attention. They are vulnerable due to several reasons, including their frail age and low economic income. Many elderly women are in poverty and living alone with the extension of life expectancy and gender mortality [5]. The traditional joint family is gradually replaced by the nuclear family, which leads to the weakening of family relations. Women live longer than their male counterparts and may no longer have a built-in caregiver [6].

The proportion of middle-aged and elderly, those aged 45 or older, is increasing in China, often with more chronic conditions than their predecessors. While the change in the age profile of the population will be accompanied by a decline in communicable diseases, an increasing number of middle-aged and elderly will raise the incidence of non-communicable diseases, placing new burdens on health care provision into the future [7]. Thus, the demand for health care services has increased, especially middle-aged and elderly females (MAEF) in rural areas. Rural females are also having lower life expectancy, compared to urban females [8,9]. Previous research has demonstrated gender differences in health care utilization, with females using more both outpatient and inpatient utilization of health care services compared to males [10]. 

The challenge of population health is gradually shifting away from traditional infectious diseases to non-communicable diseases related to an aging population, such as chronic non-communicable diseases. Most middle-aged and older women have lower levels of health, high proportion of chronic non-communicable diseases, long-duration illnesses, and high demand for inpatient services. Thus, high pressure on hospitalization expenses makes it very prone to poverty due to due to illness, which places tremendous pressure on the medical security of middle-aged and elderly women in rural China. In the United States, about three-quarters of total health care costs are related to the treatment of chronic diseases, and chronic diseases is an important factor that impacts the use of inpatient services [11]. 

The economic income of MAEF in economically underdeveloped areas is generally lower than that of men, and their social status is also relatively low. Any catastrophic medical expenses will cause serious illness and poverty due to illness in the middle-aged and elderly women, which will inevitably affect their use of inpatient services and limit their needs for inpatient service [12]. In 2003, the new rural cooperative medical system (NCMS) was launched by the Chinese central government in order to provide health insurance for China’s rural population. The NCMS is characterized by voluntary enrollment, individual and governmental co-financing, and pooled resources at the county level [13]. NCMS policy was also found to increase the utilization of health care services [14].

One important factor in this choice of focus is that middle-aged and elderly females is the key group whose utilization of inpatient services needs more attention as their health decays with age. The middle-aged and elderly female demographic faces a great disease burden. Maintaining this age-based separation in this study is thus a logical step to take.

This paper examines the utilization of inpatient services and its influencing factors for middle-aged and elderly rural females in underdeveloped regions. This study analyzes whether the utilization of inpatient service is influenced by demographic or socioeconomic factors or not, such as medical insurance (NCMS), age, gender, marital status, education, occupation, labor force, chronic disease, and income level. The findings from this study form a certain scientific basis for further modulation of the medical security system, which will allow for the development of effective corresponding countermeasures to improve the utilization of medical care, elevate health statuses, and impel the equality and accessibility of health service utilization.

## 2. Materials and Methods 

### 2.1. Subjects and Methods

Jiangxi Province is a less developed region located in middle China. This study was initially carried out in Wuyuan, Xiushui, and Luxi—three counties of Jiangxi Province—which are the first batch and second batch of new rural cooperative medical pilot counties. Multi-stage stratified cluster random sampling method was used to randomly select the research objects in the sample counties. For each of the target counties, all the townships and towns were ranked based on annual per capita income of farmers. The three-point system is used to divide the famers into three layers by the percentile method at 33.33% and 66.67% by accumulating the agricultural population in each of the target counties. The sampling interval between each layer is obtained through calculation of the total agricultural population. A random number was extracted from the random number table, and the townships and towns in the first layer were determined according to the cumulative agricultural population. The second random number was determined according to the sampling interval, and the sample towns on the second layer were selected. The third random number was determined in turn according to the sampling interval, and the sample towns on the third layer were selected. The sampling villages were selected in accordance with the sampling method of sample towns, and a total of 9 sample townships and towns were selected. Then, three administrative villages were randomly selected for each sample township (town) and a total of 27 sample villages were selected. 

**Figure 1:**
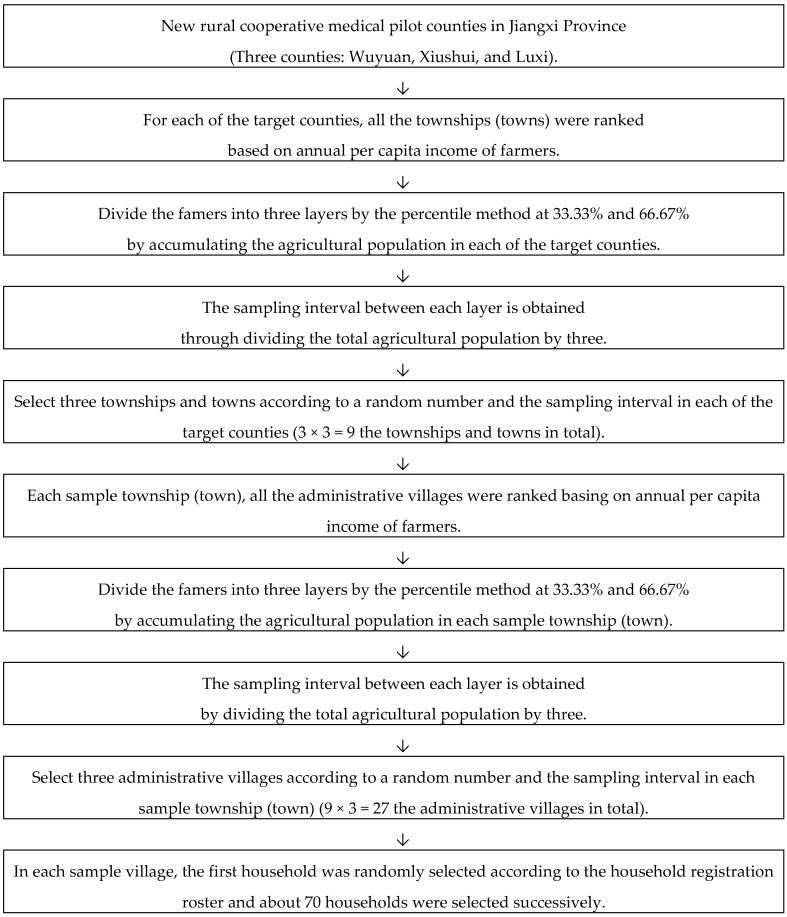
The sampling process.

In each sample village, the first household was randomly selected according to the household registration roster and about 70 households were selected successively. All household members in the family were investigated through an interview questionnaire. In this study, a number of cross-sectional surveys were conducted in 2006, 2008, 2010, 2012, and 2014. In order to ensure data comparability, the sample villages selected for the five surveys were all the same [15]. The sampling process is shown in Figure 1.

A one-to-one household survey method was used in this study. The investigator conducted a questionnaire by asking the head of the household. If the household head was not at home, the questionnaire was completed asking other members of the family who were over 18 years of age. The questionnaire included general demographic characteristics, health statuses of family members, and family members’ health service needs and utilization status [16]. The subjects in this paper were the female aged 45 and above, living in rural areas for more than half a year which were adhered to classification standard of middle-aged and elderly age by the United Nations WHO.

### 2.2. Sample Size Calculation

The sample content required in this study is formulated as follows:

(1) $$n=\frac{u_{\alpha /2}^{2}\pi \left(1-\pi \right)}{\delta^{2}}$$

Using the previous baseline results, the hospitalization rate of rural residents within one year was π = 4.00%, α=0.05, $u_{0.05/2}$, δ=0.01, with the result of: n=1308 people. There were 8082, 8015, 7524, 7857, and 7808 objects, respectively, in the five follow-up surveys, which were all larger than the theoretical sample size.

### 2.3. Weighing Methods

**Figure 2:**
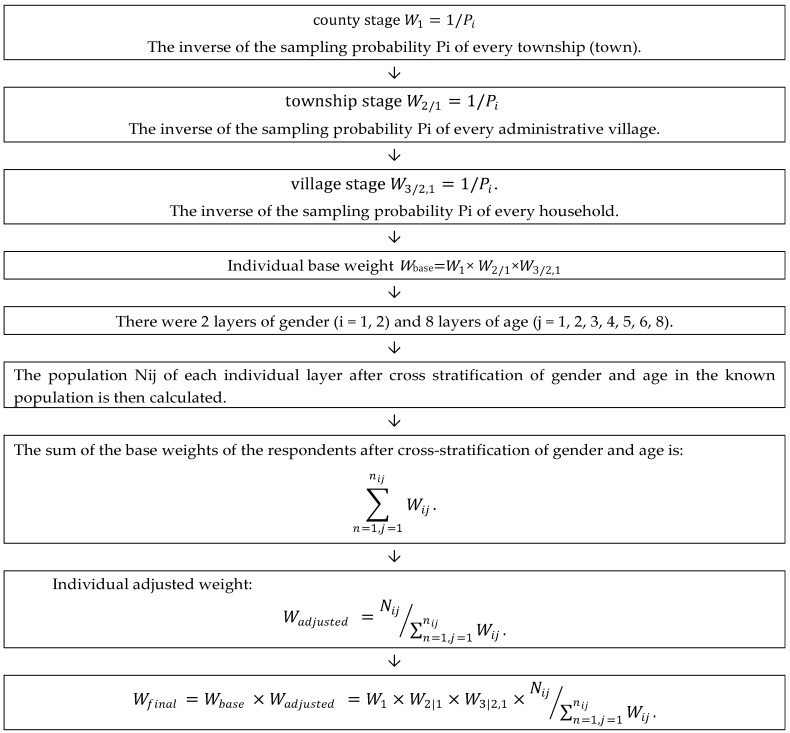
The weighted process.

Complex sampling survey refers to the random sampling of research objects in each layer according to the proportion after stratification of subjects. Owing to the existence of sampling error, there is always a deviation in population conjectured by sample, which skews the result away from features of the population. Therefore, post-stratification weighting is needed to adjust the data and reduce errors [17]. The weights were calculated in this study by first calculating the individual basis weights, then calculating the individual adjustment weights, and finally calculating the product of the individual basis weights and the adjustment weights to obtain the individual final weights [18], according to the method described previously [19]. The Weighted process is shown in Figure 2.

#### 2.3.1. The Calculation of Individual Base Weight

The sampling weight (*Wi*) of observing individual (*i*) is the inverse of the sampling probability P_i_ of that individual, i.e., *W_i_* = 1/*P_i_*. Therefore, for three-stage stratified cluster random sampling, the sampling weight of the sampling units in stage 1 is *W*_1_, the sampling weight of the sampling units in stage 2 is *W*_2| 1_, and the sampling weight of the sampling units in stage 3 is *W*_3| 2, 1_. The individual base weight is the product of sampling weights of each stage, so the individual base weight observation units is: *W_base_* = *W*_1_ × *W*_2| 1_ × *W*_3| 2, 1_.

#### 2.3.2. The Calculation of Individual Adjusted Weight

The adjustment weight is calculated to make the distribution of individual sex and age in the sample consistent with the distribution of individual sex and age in the known population. First, the gender and age are stratified, that is, there were 2 layers of gender (*i* = 1, 2) and 8 layers of age (*j* = 1, 2, 3, 4, 5, 6, 8). The overlapping of these layers is the minimum layer, with a total of 16 layers (2 × 8 = 16). The population *N_ij_* of each individual layer after cross stratification of gender and age in the known population is then calculated.

The sum of the base weights of the respondents after cross-stratification of gender and age is:

(2) $$\overset{n_{ij}}{\underset{n=1,j=1}{\sum }}W_{ij}.$$

Individual adjusted weights in each layer after cross-stratification of gender and age in the sample is calculated:

(3) Wadjusted=Nij∑n=1,j=1nijWij.

#### 2.3.3. The Calculation of Individual Final Weight

Individual final weight is the product of the individual basis weight and the individual adjustment weight, and the calculation formula is:

(4) Wfinal=Wbase×Wadjusted=W1×W2|1×W3|2,1×Nij∑n=1,j=1nijWij.

### 2.4. Index Construction

#### 2.4.1. Chronic Diseases

Various chronic diseases, including chronic infectious and non-communicable diseases, were clearly diagnosed by medical staff through inquiring surveyed people during the first half year of the survey; participants who were diagnosed with chronic diseases by doctors half a year before the survey, and had seizures and were taking treatment, including medication and physical therapy, was also taken into account.

#### 2.4.2. Hospitalization within One Year

Surveyed people went to the hospital for hospitalization within one year from the date of survey.

#### 2.4.3. Should be Hospitalized but Not Hospitalized

Within one year from the date of the survey, the respondent had a case in which the doctors recommended the person to be hospitalized but was not actually hospitalized.

#### 2.4.4. Early Hospital Discharge

A case of early discharge from hospital in the previous year from the date of investigation.

#### 2.4.5. Utilization of Inpatient Services

The hospitalization rate, early discharge rate, and rate of recommendation for hospitalization but not hospitalized in the past year. Hospitalization rate (%) = number of hospitalizations/number of surveyed ×100%; Early discharge rate (%) = number of patients discharged in advance/number of hospitalization ×100%; rate of supposed to be hospitalized but not hospitalized (%) = number of recommended but not hospitalized/number of recommended for hospitalization but not hospitalized + number of hospitalized inpatients×100%.

#### 2.4.6. Income Level

The household income per capita was arranged in order of low to high, and then divided into five groups: low level income, low middle level income, middle level income, upper middle level income, and high-level income.

#### 2.4.7. The New Rural Cooperative Medical Care (NRMC)

This is a new type of rural medical care and mutual aid system that is organized, guided, and supported by the government. The NRMC system collects funds through individual payment, collective support, and government subsidy with voluntary participation by farmers, and the overall goal is to better coordinate major diseases and reduce rural medical disasters [13,20,21,22,23]. The NRMC started its pilot implementation in 2003 and the amount of funds raised in 2006 was 30 yuan in Jiangxi Province. The level of fundraising increased to 60 yuan in 2006, 300 yuan in 2012, and reached 390 yuan by 2014, respectively. At the same period, the hospitalization deduction line was designed to be 100 yuan for the designated township (town) level medical institutions with the hospitalization reimbursement ratio of 60%. The initial deduction line was removed in 2014 for the designated medical institutions and the proportion of hospitalization reimbursement increased to 90% (Table 1).

### 2.5. Statistic Methods

Epidata 3.0 was employed to input data, and the database was imported into Excel and transferred to SPSS 24.0 statistical software (IBM Corporation, Chicago, IL, USA,) for analysis. The differences between variables were compared using the χ^2^ statistic method after the complex sampling was weighted. The complex sampling logistics regression was used in multi-factor analysis with the test standard set as α = 0.05 and to analyze 3 index factors in the utilization of inpatient services. Crude odds ratio (cOR) refers to the result obtained by putting variables into the equation separately for analysis. Adjust odds ratio (aOR) refers to the result of putting the independent variables into thee equations together for analysis [24,25].

### 2.6. Quality Control

In order to ensure the comparability of the data, both the follow-up survey and the baseline survey were sampled in the same towns and villages. Study team members included postgraduates of the public health school of Nanchang University and they all had experience in survey study and received special training before the investigation including unified standard language. During the survey, village cadres of the study sites were available to assist investigators in any translation and completion of the survey together. At the end of the survey day, all investigators gathered together to input data. If any errors or omissions were found, the local village cadres were contacted to reach the respondents by telephone or other approaches to complete the questionnaire. Data input was carried out through double entry with logical correction and timely error correction.

The Maye index is an indicator for judging the quality of survey data by evaluating the logic within the survey data [26]. A Maye coefficient greater than 60 indicates that there is a serious age preference or accumulation in the survey population data. The Maye index is less than 60, which can be judged as good data quality [27]. The Maye index of the six surveys in this study was 7.78, 9.81, 12.02, 8.52, 3.54, and 7.07, respectively, indicating that there was no serious age preference in the sample population data, and the survey data quality was good.

The goodness-of-fit test is a statistical method developed by K Pearson, a British statistician, and it is used to ensure the consistency between actual observations and theoretical numbers, calculated according to some hypothesis or model [28]. This study used the data of the population age (gender) of the Jiangxi Statistical Yearbook as a reference and the census data of each year in Jiangxi Province as the theoretical frequency, which were assumed to be in a normal distribution. Each survey data was used as sample data to observe the degree to which the distribution of sample data fits the theoretical frequency distribution. If there was no significant difference in the test results, it indicated that the survey data are of good quality and the sample data are representative. The chi-square values of the population age composition of Jiangxi Province for the six-time surveys were 3.17, 4.99, 7.48, 5.63, 6.44, and 3.49, respectively. The chi-square values of the gender composition fitness test were 0.001445, 0.000196, 0.000484, 0.110294, 0.002118, and 0.006895, respectively. All the P values are greater than 0.05, which indicates that the distribution of age and gender of the sample data is not significantly different from the overall population distribution in Jiangxi Province, and thus the survey data is representative.

### 2.7. Ethical Approval

The study was approved by the Nanchang University Institutional Review Board. We have ethical approval on file. Oral consents of farmers who participated in the survey were obtained. 

## 3. Results

### 3.1. Sampling Features

Before the data weighting, there were 6230 MAEF selected in five surveys from 2006 to 2014, including 1151, 1220, 1180, 1301, and 1378, respectively. After the data weighting, the group aged between 45–54 accounted for the highest proportion with 46.4%, and the group aged 75 and over had the lowest proportion with 8.6%. Among these MAEF, 54.7% were primary-school educations, 81.7% were married, 82.2% were farmers, 63.1% were attributed to the labor force, and 23.8% suffered from chronic diseases. As shown in Table 2, there is no statistical difference between different years, different age groups, different levels of education, and marital status.

### 3.2. The Change of Inpatient Service Utilization of Middle-Aged and Elderly Females

The hospitalization rate of MAEF was 6.7% in 2006 and increased to 14.2% in 2014. The rate for those females who were supposed to be hospitalized but not hospitalized was as high as 30.3% in 2006 and dropped to 13.2% in 2014. The rate of early discharge in this female group was 23.7% in 2006 and fell to 8.6% in 2014. As shown in Table 3, there were statistically significant differences in hospitalization rates, the rates for recommended hospitalization but not hospitalized females, and early discharge rates among these women in different years (*p* < 0.05).

### 3.3. The Univariate Analysis of Inpatient Service Utilization of Middle-Aged and Elderly Females

The hospitalization rate of MAEF was 10.0%, and the rate for those who were supposed to be hospitalized but were not hospitalized was 19.2%, and the early discharge rate was 10.6%. There were significant differences in the hospitalization rates among middle-aged and older women with different demographic characteristics, including ages, education level, occupation, labor, and chronic disease (*p* < 0.05). The hospitalization rate of the 65–74 years old group, illiterate group, married group, farmer group, non-labor group, and chronic disease group was higher than other groups, accounting for 13.4%, 13.2%, 10.0%, 11.0%, 14.7%, and 21.2%, respectively. There was no statistical difference in the rate of early discharge of MAEF with different demographic characteristics (*p* > 0.05). There was a statistically significant difference in the rate of hospitalization between (*p* > 0.05). The rates of hospitalization for patients who were divorced or widowed or those with chronic diseases who should be hospitalized but not hospitalized were 28.2% and 29.1%, higher than that of other groups (Table 3).

### 3.4. The Multi-Factor Analysis of Inpatient Service Utilization of Middle-Aged and Elderly Females

Table 4 shows the results of the complex sampling logistics regression analysis. This analysis was conducted by taking three factors as dependent variables and eight other factors as independent variables. The dependent variable includes the status of hospitalization, recommended for hospitalization but not hospitalized, and early discharge from hospital. On the other hand, independent variable considers factors like year, age group, education level, marital status, occupation, chronic diseases, income level, and whether she’s in the labor force or not. It is preferable to analyze after transforming the categorical variable into dummy variables. The variables assignment summary for complex sampling logistic regression analysis is shown in Table 5. First, the independent variables were put into the equation individually for analysis, and the crude odds ratio (cOR) of each independent variable was calculated. Then, the independent variables were put into the equation for analysis, and the adjustment odds ratio (aOR) of each independent variable was calculated. The complex sampling logistics regression analysis of hospitalization of MAEF revealed that the rates of hospitalization in 2012 (aOR = 2.094, *p* < 0.05) and 2014 (aOR = 2.384, *p* < 0.05) were higher than that in 2006. MAEF with chronic diseases are more likely to be hospitalized (aOR = 3.682, *p* < 0.05). The probability of hospitalization in divorced or widowed MAEF is lower (aOR = 0.177, *p* < 0.05). The analysis also shows that non-labor females have a higher probability of getting hospitalized (aOR = 1.976, *p* < 0.05). The rates of middle-aged and elderly females who should be hospitalized but not hospitalized in 2012 (aOR = 0.107, *p* < 0.05) and 2014 (aOR = 0.177, *p* < 0.05) were lower than that in 2006. The MAEF with chronic diseases are more likely to become not hospitalized while they should be (aOR = 3.258, *p* < 0.05). The early discharge rates of NAEF in 2012 (aOR = 0.046, *p* < 0.05) and 2014 (aOR = 0.347, *p* < 0.05) were lower than that in 2006. Divorced or widowed MAEF are more likely to get an early discharge from hospital (aOR = 3.237, *p* < 0.05) and those with chronic diseases are even more likely to get an early discharge from hospital (aOR = 7.689, *p* < 0.05) (Table 4).

## 4. Discussion 

Jiangxi Province, as one of the economically underdeveloped regions, has a large number of labor force output. However, middle-aged and elderly females who do not have many labor skills have to stay in the countryside to undertake agricultural production and heavy and trivial housework. Therefore, they become the vulnerable group whom needs to get special attention. In the past 20 years, China has carried out a series of medical and health security system reforms in order to promote health equity and eliminate the gap between groups; medical coverage has almost reached the entire population [29]. However, there are few studies investigating the utilization of inpatient services in vulnerable groups. This study found that more than half of MAEF staying in rural areas were over 55 years old, the majority (75%) had primary education or below, 94.6% were farmers, and 25.7% suffered from chronic diseases. Due to the low health and high ratio of chronic diseases among MAEF, the utilization of inpatient services is worthy of attention.

The hospitalization rate of middle-aged and elderly rural females in underdeveloped regions has an overall upward trend from 2006 to 2014. In 2006, the proportion of inpatients for those who should be hospitalized but not hospitalized exhibited an overall downward trend. A similar trend is also detected for the early discharge rate. According to the study by the China Health and Retirement Longitudinal Study (CHARLS), about 9% of middle-aged and elderly people have been hospitalized in the past year, which means at least 40.4 million middle-aged and elderly people are hospitalized in China every year [4]. According to the fourth national health service survey of China, the hospitalization rate of the whole rural population was 9.0% in 2013, varying between 7.6% and 10.0% in different regions [30], which was lower than the survey results of this study. This can be explained that the use of inpatient services for middle-aged and elderly females in rural areas in less developed areas in China such as Jiangxi has been gradually enhanced since 2006.

The medical security system is known to have an impact on the use of inpatient services [23,31,32]. Some studies have shown that patients enrolled in NRCM are more likely to seek health care services [2,33,34,35]. The NRCM got started in Jiangxi Province in 2003, and Wuyuan, Xiushui, and Luxi counties were randomly selected for this study to examine the impact of this new system in 2005. The rate of hospitalization of middle-aged and elderly females in 2014 was higher than that in 2006 (aOR = 2.384), while the early discharge rate from hospital (aOR = 0.177) and rate of females who should be hospitalized but not hospitalized (aOR = 0.347) were lower than those reported in 2006. These findings indicate that the utilization of inpatient services for middle-aged and elderly females in rural underdeveloped areas was further promoted with time. The positive change may be related to the improvement of the NRCM system [36]. In addition, with the increase of finding levels from 2006 to 2014, its reimbursement compensation program is also constantly being adjusted and improved [31]. In 2014, the township (town)-level fixed-point medical institutions did not set up deductible lines, and the proportion of hospitalization compensation increased to 90%. The proportion of hospitalization compensation for designated medical institutions at county (city, district)-level reached 80%. These results reveal that the improvement of compensation programs has improved residents’ utilization of primary inpatient medical services. Also the implementation of the NRCM system has effectively provided health protection for middle-aged and elderly women, and promoted the utilization of inpatient health services for rural middle-aged and elderly women in underdeveloped areas [3].

Present studies have shown that household income is beneficial to the use of inpatients in the rich, which is the dominant determinant of inequality in the utilization of inpatient services since more wealthy individuals are able to pay for and utilize more inpatient services [37,38,39]. But some studies have shown that the differences of health service utilization between income groups were not significant [40]. In this study, the average annual household income was divided into five levels and the differences in the inpatient service utilization among middle-aged and elderly females at different levels were not statistically significant. Some studies have shown that residents’ household economic income level should play a role in their utilization of hospitalization services, but this study did not find that household economic income has a significant impact on the utilization of hospitalization services for middle-aged and elderly females. From 2006 to 2014, China’s economy developed rapidly, and the per capita annual income of residents also greatly improved. Our study also reveals that the gradual increase in the utilization of inpatient service among MAEF is not affected by economic income, but mainly due to the continuous improvement of the new rural cooperative medical insurance system. The new rural cooperative medical care system is a basic medical insurance system established by China in the world’s largest population group. The establishment of the system greatly facilitated the demand and utilization of medical services by rural residents, improved the fairness and access to medical service in rural areas, and offered special benefit to vulnerable groups [41] such as middle-aged and elderly women. At present, this system has been merged with the basic medical care system for urban residents and is constantly being improved [31]. Findings from this study suggest to speed up the improvement of this medical security system, further improve its financing level and compensation scheme, so as to provide better medical protection for urban and rural residents [12,42].

Many studies have shown that the utilization of inpatient service is influenced by demographic or socioeconomic factors, such as age, gender, marital status, education, medical insurance, standard of living, and urban residence [4,29,43,44]. Based on the multivariate complex sampling logistic regression analysis, this study shows that the utilization of inpatient services of middle-aged and elderly females in underdeveloped areas was mainly related to marital status, whether they were in the labor force, and whether they suffered from chronic diseases. This study also reveals that, compared to married MAEF, divorced or widowed ones inadequately utilized hospitalization services, with a lower likelihood of hospitalization (aOR = 0.569), but higher rate for the recommended hospitalization but hot hospitalized (aOR = 3.237). It may be due to divorced and widowed females who need to bear the pressure of life alone and have a heavier living burden. In situations where a doctor recommends hospitalization, divorced or widowed ones may choose to self-medicate or postpone hospitalization. The lack of family care during hospitalization may also affect the utilization of hospitalization services for middle-aged and elderly females who are divorced or widowed.

In this study, it was found that the hospitalization rate of middle-aged and elderly females not in the labor force was 1.976 times than that of elderly females in the labor force (*p* < 0.05). It is possible that non-labor MAEF in rural areas are older with poorer health [45].

This study shows that the middle-aged and elderly females with chronic diseases had a higher rate for hospitalization (aOR = 3.682), higher rate for recommended hospitalization but not hospitalized (aOR = 7.689), and higher rate for early discharge (aOR = 3.258) than those without chronic diseases. This may reflect the situation in that MAEF with chronic non-communicable diseases have the needs for health care on one hand, however, they have heavier medical economic burden because of long-time illness and the lack of the sense of paying attention to diseases. In addition, as the NRCM can only recapture some of the cost for medicine which may aggravate more cases of early hospital discharge and also the recommended hospitalization but not hospitalized [46,47]. These findings indicate an urgent need to strength the prevention and treatment of chronic diseases among MAEF and improve the health status of these females [48]. Also, there is a need to design appropriate corresponding compensation scheme for patients with chronic diseases, so that they can make full use of inpatient services.

## 5. Limitation

Data in this paper is collected according to the sampling plan using multi-stage stratified cluster sampling with the assistance of the local government department staff and village cadres through interviews with householders. Although every household answered the questionnaire to avoid bias caused by not replying, the use of the local government department staff and village cadres may cause selection bias. Thus, more attention was paid to limit and reduce the involvement of local government staff and village cadres. 

The annual per capita income included in this study may have recall bias, which is a common limitation of the survey research. Our research group conducted a special explanation for the survey method of annual per capita income. All investigators followed the same standard for the recording of household per capita income thereby reducing error. In this study, the utilization of inpatient services was answered only by the respondents, and some villagers would even present their hospitalization documents to ensure the accuracy of information.

One of the limitations of this study was that household heads were responsible for answering all the questions for their household members. In the absence of the household head, an adult member of the household with information about the household members responded for the household members. It may be problematic for the household head or the representative to provide accurate responses for all household members. However, considering that the household sizes are generally small (often less than five), and being a rural area where information is often shared among household members, we do not expect so much variation in the responses from individuals from the same household. And the utilization of inpatient services of household members in rural regions is significant, which may get accurate responses.

## 6. Conclusions

In the rural regions of China’s economically underdeveloped areas, the continuous improvement of the NRCM system has promoted the utilization of inpatient services among MAEF, and alleviates the economic burden of hospitalization of some middle-aged and elderly women. Therefore, the medical security system should be further improved and implemented in underdeveloped regions in the future. Since the hospitalization rate is significantly higher in middle-aged and elderly females with chronic diseases than in those without chronic diseases, it is important and necessary to strengthen the prevention and treatment of chronic diseases among middle-aged and elderly women and also elevate their health status in the future.

## Figures and Tables

**Table 1:** Financing level and compensation ratio of new rural cooperative medical care from 2006 to 2014.

Variable	2006	2008	2010	2012	2014
NRMC annual fundraising level per person (¥)	60	100	150	300	390
Annual fee per person (¥)	10	20	30	50	70
Financial subsidy per person per year (¥)	50	80	120	250	320
Hospitalization reimbursement rates (%)					
Township (town) level designated hospital	60	60	80	90	90
County level designated hospital	50	50	65	75	80
County level above designated hospital	40	40	45	60	50
County level above non-fixed hospital outside the county	30	30	35	50	35

NRMC: The new rural cooperative medical care.

**Table 2:** Distribution of demographic characteristics of MAEF in three counties between 2006 and 2014 (%).

Variable	2006	2008	2010	2012	2014	Total
Sample (N)	1151	1220	1180	1301	1378	6230
Weighted Number	120,419	142,635	147,380	153,333	200,397	764,164
Age (year)						
45~	47.8	46.7	48.5	47.5	43.0	46.4
55~	26.0	29.1	29.0	27.9	30.8	28.8
65~	16.4	15.7	15.1	16.7	16.6	16.1
75~	9.8	8.5	7.3	7.8	9.6	8.6
Educational level						
Illiterate	23.9	16.5	14.7	10.7	21.5	17.4
Elementary school	56.2	54.3	53.4	56.8	53.5	54.7
Middle school and above	19.9	29.2	32.0	32.5	25.0	27.8
Marital status						
Married	79.0	82.8	82.3	81.5	82.2	81.7
Divorced/widowed	21.0	17.2	17.7	18.5	17.8	18.3
Occupation ^a^						
Non-farmer	3.4	6.1	64.9	5.4	5.4	17.8
Farmer	96.6	93.9	35.1	94.6	94.6	82.2
Labor force ^a^						
No	42.0	53.8	30.9	27.1	33.6	36.9
Yes	58.0	46.2	69.1	72.9	66.4	63.1
Chronic disease ^a^						
No	80.0	77.5	69.9	80.5	74.3	76.2
Yes	20.0	22.5	30.1	19.5	25.7	23.8
Income level (¥)						
Low level	1631.00	1303.18	2753.79	3415.98	3552.92	2606.76
Middle low level	2834.72	2526.63	4647.01	5874.59	8466.74	5189.30
Middle level	3878.76	3529.25	6130.16	7999.18	11,565.42	7120.34
Middle high level	5265.60	4745.82	7782.53	10,504.63	15,640.11	9697.77
High income level	10,115.62	8362.40	12,110.45	19,438.26	40,911.26	20,201.21

^a^ chi-square statistic between different years *p* < 0.005.

**Table 3:** Comparison of the difference of three indexes of inpatient service utilization after data weighted between different characteristic MAEF (%).

Demographic Characteristics	The Rate of Hospitalization	The Rate of Early Discharge	The Rate of Hospital Avoidance
Year			
2006	6.7	23.7	30.3
2008	9.3	12.2	35.2
2010	6.4	11.7	24.0
2012	11.0	5.9	2.3
2014	14.2	8.6	13.2
*p* ^a^	0.003	0.023	<0.001
Age (year)			
45~	7.0	15.7	19.7
55~	12.1	11.1	20.9
65~	13.4	4.7	15.7
75~	12.6	5.6	19.0
*p* ^a^	0.022	0.099	0.714
Educational level			
Illiterate	13.2	11.7	23.9
Elementary school	10.0	9.8	18.1
Middle school and above	7.9	11.7	16.8
*p* ^a^	0.018	0.870	0.378
Marital status			
Married	10.0	10.8	16.9
Divorced/widowed	9.7	9.7	28.2
*p* ^a^	0.837	0.754	0.008
Occupation			
Non-farmer	5.3	4.0	15.5
Farmer	11.0	11.4	19.6
*p* ^a^	0.007	0.056	0.546
Labor force			
No	14.7	8.4	22.8
Yes	7.2	13.3	14.6
*p* ^a^	<0.001	0.175	0.185
Chronic diseases			
No	6.4	7.2	6.3
Yes	21.2	14.1	29.1
*p* ^a^	<0.001	0.125	<0.001
Income level			
Low level	10.7	10.8	25.3
Middle low level	11.0	10.0	16.0
Middle level	10.4	4.7	16.8
Middle high level	9.6	14.1	18.2
High income level	7.8	14.2	16.7
*p* ^a^	0.303	0.540	0.498
Total	10.0	10.6	19.2

^a^ Pearson Chi-Square test.

**Table 4:** Analysis three indexes of inpatient service utilization using complex sampling logistic regression among mid-aged and older females (%, 95% CI).

	Hospitalization		Early Discharge		Hospital Avoidance	
	cOR	aOR	cOR	aOR	cOR	aOR
Year	(2006 as Ref.)	(2006 as Ref.)	(2006 as Ref.)	(2006 as Ref.)	(2006 as Ref.)	(2006 as Ref.)
2008	1.432 (0.720, 2.849)	1.312 (0.682, 2.524)	0.447 (0.242, 0.826) *	0.249 (0.083, 0.750) *	1.246 (0.830, 1.870)	1.156 (0.761, 1.757)
2010	0.956 (0.530, 1.727)	1.016 (0.565, 1.829)	0.424 (0.130, 1.388)	0.497 (0.142, 1.737)	0.726 (0.408, 1.291)	0.563 (0.250, 1.265)
2012	1.729 (1.304, 2.293) *	2.094 (1.509, 2.907) *	0.202 (0.055, 0.739) *	0.107 (0.054, 0.209) *	0.054 (0.017, 0.172) *	0.046 (0.013, 0.156) *
2014	2.325 (1.307, 4.134) *	2.384 (1.337, 4.252) *	0.303 (0.109, 0.846) *	0.177 (0.072, 0.437) *	0.351 (0.194, 0.636) *	0.347 (0.154, 0.782) *
Age	(45~ as Ref.)	(45~ as Ref.)	(45~ as Ref.)	(45~ as Ref.)	(45~ as Ref.)	(45~ as Ref.)
55~	1.832 (1.132, 2.965) *	1.387 (0.748, 2.572)	0.672 (0.359, 1.257)	0.611 (0.321, 1.162)	1.073 (0.628, 1.834)	0.694 (0.396, 1.217)
65~	2.059 (1.456, 2.911) *	1.406 (0.983, 2.012)	0.264 (0.062, 1.121)	0.174 (0.024, 1.253)	0.760 (0.334, 1.730)	0.339 (0.154, 0.745)
75~	1.920 (0.959, 3.844)	1.456 (0.634, 3.342)	0.318 (0.100, 1.014)	0.322 (0.084, 1.240)	0.955 (0.413, 2.208)	0.455 (0.185, 1.123)
Educational level	(Illiterate as Ref.)	(Illiterate as Ref.)	(Illiterate as Ref.)	(Illiterate as Ref.)	(Illiterate as Ref.)	(Illiterate as Ref.)
ES	0.732 (0.546, 0.981) *	0.900 (0.687, 1.178)	0.817 (0.309, 2.162)	0.784 (0.292, 2.110)	0.704 (0.381, 1.302)	1.153 (0.504, 2.636)
MSA	0.569 (0.360, 0.898) *	0.784 (0.495, 1.241)	1.001 (0.267, 3.747)	0.566 (0.131, 2.444)	0.643 (0.300, 1.379)	0.609 (0.238, 1.558)
Marital status	(Married as Ref.)	(Married as Ref.)	(Married as Ref.)	(Married as Ref.)	(Married as Ref.)	(Married as Ref.)
Divorced/widowed	0.968 (0.689, 1.359)	0.569 (0.399, 0.811) *	0.885 (0.385, 2.033)	1.963 (0.708, 5.446)	1.926 (1.222, 3.036) *	3.237 (1.397, 7.500) *
Occupation	(Non-farmer as Ref.)	(Non-farmer as Ref.)	(Non-farmer as Ref.)	(Non-farmer as Ref.)	(Non-farmer as Ref.)	(Non-farmer as Ref.)
Farmer	2.181 (1.271, 3.743) *	1.356 (0.909, 2.023)	3.086 (0.909, 10.476)	7.750 (0.985, 60.993)	1.333 (0.485, 3.665)	1.683 (0.523, 5.418)
Labor force	(Yes as Ref.)	(Yes as Ref.)	(Yes as Ref.)	(Yes as Ref.)	(Yes as Ref.)	(Yes as Ref.)
No	2.208 (1.527, 3.192) *	1.976 (1.373, 2.846) *	1.676 (0.763, 3.680)	0.471 (0.187, 1.188)	1.729 (0.736, 4.061)	1.140 (0.442, 2.943)
Chronic disease	(No as Ref.)	(No as Ref.)	(No as Ref.)	(No as Ref.)	(No as Ref.)	(No as Ref.)
Yes	3.913 (3.095, 4.948) *	3.682 (2.941, 4.610) *	2.113 (0.773, 5.775)	3.258 (1.142, 9.299) *	6.065 (2.743, 13.408) *	7.689 (3.113, 18.987) *
Income level	(Low income as Ref.)	(Low income as Ref.)	(Low income as Ref.)	(Low income as Ref.)	(Low income as Ref.)	(Low income as Ref.)
Low	1.032 (0.753, 1.415)	1.080 (0.825, 1.415)	0.917 (0.198, 4.242)	1.123 (0.225, 5.607)	0.563 (0.250, 1.267)	0.690 (0.280, 1.700)
Middle	0.975 (0.627, 1.515)	1.108 (0.735, 1.671)	0.408 (0.113, 1.475)	0.437 (0.093, 2.050)	0.596 (0.294, 1.206)	0.616 (0.212, 1.788)
Middle high	0.891 (0.728, 1.091)	1.078 (0.821, 1.415)	1.359 (0.512, 3.606)	1.846 (0.652, 5.223)	0.655 (0.210, 2.043)	0.879 (0.253, 3.054)
High	0.713 (0.538, 0.946) *	0.976 (0.729, 1.307)	1.371 (0.384, 4.886)	1.186 (0.413, 3.408)	0.591 (0.247, 1.415)	0.557 (0.194, 1.595)

* *p* < 0.05.

**Table 5:** The variable assignment summary for complex sampling logistic regression analysis.

Factors	Variable Name	Factor Assignment
Dependent variables		
Hospitalization	Y_1_	1 = Yes,0 = No (reference)
Hospital avoidance	Y_2_	1 = Yes,0 = No (reference)
Early discharge	Y_3_	1 = Yes,0 = No (reference)
Independent variables		
Year	X_1_, X_2_, X_3_, X_4_	2006 (reference): X_1_ = 0, X_2_ = 0, X_3_ = 0, X_4_ = 0
		2008: X_1_ = 1, X_2_ = 0, X_3_ = 0, X_4_ = 0
		2010: X_1_ = 0, X_2_ = 1, X_3_ = 0, X_4_ = 0
		2012: X_1_ = 0, X_2_ = 0, X_3_ = 1, X_4_ = 0
		2014: X_1_ = 0, X_2_ = 0, X_3_ = 0, X_4_ = 1
Age (year)	X_5_, X_6_, X_7_	45~(reference): X_5_ = 0, X_6_ = 0, X_7_ = 0
		55~: X_5_ = 1, X_6_ = 0, X_7_ = 0
		65~: X_5_ = 0, X_6_ = 1, X_7_ = 0
		75~: X_5_ = 0, X_6_ = 0, X_7_ = 1
Educational level	X_8_, X_9_	Illiterate (reference): X_8_ = 0, X_9_ = 0
		Elementary school: X_8_ = 1, X_9_ = 0
		Middle school and above: X_8_ = 0, X_9_ = 1
Marital status	X_10_	0 = Married (reference); 1 = divorced/widowed
Occupation	X_11_	0 = Non-farmer (reference); 1 = Farmer
Labor force	X_12_	0 = Yes (reference); 1 = No
Chronic diseases	X_13_	0 = No (reference); 1 = Yes
Income level	X_14_, X_15_, X_16_, X_17_	Low level income (reference): X_14_ = 0, X_15_ = 0, X_16_ = 0, X_17_ = 0
		Low middle level income: X_14_ = 1, X_15_ = 0, X_16_ = 0, X_17_ = 0
		Middle level income: X_14_ = 0, X_15_ = 1, X_16_ = 0, X_17_ = 0
		Upper middle level income: X_14_ = 0, X_15_ = 0, X_16_ = 1, X_17_ = 0
		High level income: X_14_ = 0, X_15_ = 0, X_16_ = 0, X_17_ = 1

## Abbreviations

MAEF	middle-aged and elderly females
NRMC	New-type Rural Cooperative Medical Scheme
cOR	Crude Odds ratio
aOR	Adjust Odds ratio

## References

[1] National Bureau of Statistics of the People’s Republic of China (2018). China Statistical Yearbook. http://www.stats.gov.cn/tjsj/ndsj/2018/indexch.htm.

[2] Wang X., He X., Zheng A., Ji X. (2014). The effects of China’s New Cooperative Medical Scheme on accessibility and affordability of healthcare services: An empirical research in Liaoning Province. BMC Health Serv. Res..

[3] Yuan B., Jian W., He L., Wang B., Balabanova D. (2017). The role of health system governance in strengthening the rural health insurance system in China. Int. J. Equity Health.

[4] Zhang C., Lei X., Strauss J., Zhao Y. (2017). Health Insurance and Health Care among the Mid-Aged and Older Chinese: Evidence from the National Baseline Survey of CHARLS. Health Econ..

[5] Ogura S., Jakovljevic M.M. (2018). Editorial: Global Population Aging—Health Care, Social and Economic Consequences. Front. Public Health.

[6] Crouch E., Probst J., Bennett K., Eberth J. (2018). Gender and geographic differences in Medicare service utilization during the last six months of life. J. Women Aging.

[7] Wang Y., Wang J., Maitland E., Zhao Y., Nicholas S., Lu M. (2012). Growing old before growing rich: Inequality in health service utilization among the mid-aged and elderly in Gansu and Zhejiang Provinces, China. BMC Health Serv. Res..

[8] Singh G.K., Siahpush M. (2014). Widening rural-urban disparities in life expectancy, U.S., 1969–2009. Am. J. Prev. Med..

[9] Kindig D.A., Cheng E.R. (2013). Even as mortality fell in most US counties, female mortality nonetheless rose in 42.8 percent of counties from 1992 to 2006. Health Aff..

[10] Bertakis K.D., Azari R., Helms L.J., Callahan E.J., Robbins J.A. (2000). Gender differences in the utilization of health care services. J. Fam. Pract..

[11] Hoffman C., Rice D., Sung H.-Y. (1996). Persons With Chronic Conditions: Their Prevalence and Costs. JAMA.

[12] Wu Y., Zhang L., Liu X., Ye T., Wang Y. (2018). Geographic variation in health insurance benefits in Qianjiang District, China: A cross-sectional study. Int. J. Equity Health.

[13] Li M., Wang C. (2017). The Association Between the New Rural Cooperative Medical System and Health Care Seeking Behavior Among Middle-Aged and Older Chinese. J. Aging Soc. Policy.

[14] Wagstaff A., Lindelow M., Jun G., Ling X., Juncheng Q. (2009). Extending health insurance to the rural population: An impact evaluation of China’s new cooperative medical scheme. J. Health Econ..

[15] Zhang L., Yuan Z., Maddock J.E., Zou J., Zheng Z., Zhou W., Zheng H. (2014). Chronic disease prevalence and influencing factors among rural residents in Jiangxi, China. Int. Health.

[16] Zou J., Yang W., Cook D.M., Yuan Z., Zhang L., Wang X. (2016). New cooperative medical financing policy and hospitalization in rural China: Multi-stage cross-sectional surveys. Int. Health.

[17] Platt R.W., Harper S.B. (2013). Survey data with sampling weights: Is there a “best” approach?. Environ. Res..

[18] Pan B., Yuan Z., Zou J., Cook D.M., Yang W. (2016). Elderly hospitalization and the New-type Rural Cooperative Medical Scheme (NCMS) in China: Multi-stage cross-sectional surveys of Jiangxi province. BMC Health Serv. Res..

[19] Pan B., Towne S.D., Chen Y., Yuan Z. (2017). The inequity of inpatient services in rural areas and the New-Type Rural Cooperative Medical System (NRCMS) in China: Repeated cross sectional analysis. Health Policy Plan..

[20] Zhang L., Cheng X., Liu X., Zhu K., Tang S., Bogg L., Dobberschuetz K., Tolhurst R. (2010). Balancing the funds in the New Cooperative Medical Scheme in rural China: Determinants and influencing factors in two provinces. Int. J. Health Plan. Manag..

[21] Zhang G., Zhang L., Wu S., Xia X., Lu L. (2016). The convergence of Chinese county government health expenditures: Capitation and contribution. BMC Health Serv. Res..

[22] Yuan S., Rehnberg C., Sun X., Liu X., Meng Q. (2014). Income related inequalities in New Cooperative Medical Scheme: A five-year empirical study of Junan County in China. Int. J. Equity Health.

[23] Yang W. (2013). China’s new cooperative medical scheme and equity in access to health care: Evidence from a longitudinal household survey. Int. J. Equity Health.

[24] Homwong N., Diaz A., Rossow S., Ciarlet M., Marthaler D. (2016). Three-Level Mixed-Effects Logistic Regression Analysis Reveals Complex Epidemiology of Swine Rotaviruses in Diagnostic Samples from North America. PLoS ONE.

[25] Rader K.A., Lipsitz S.R., Fitzmaurice G.M., Harrington D.P., Parzen M., Sinha D. (2017). Bias-corrected estimates for logistic regression models for complex surveys with application to the United States’ Nationwide Inpatient Sample. Stat. Methods Med Res..

[26] Mills S. (1979). Quality control sampling. Health Care Newsl..

[27] Xie F., Jiang X., Yuan F., Chen X., Yuan Z., Lu Y. (2018). Impact of the New Cooperative Medical Scheme on the Rural Residents’ Hospitalization Medical Expenses: A Five-Year Survey Study for the Jiangxi Province in China. Int. J. Environ. Res. Public Health.

[28] Efendi A., Drikvandi R., Verbeke G., Molenberghs G. (2017). A goodness-of-fit test for the random-effects distribution in mixed models. Stat. Methods Med. Res..

[29] Wang Z., Li X., Chen M., Si L. (2018). Social health insurance, healthcare utilization, and costs in middle-aged and elderly community-dwelling adults in China. Int. J. Equity Health.

[30] National Health Council (2018). China Health Statistics Yearbook.

[31] Wang X., Zheng A., He X., Jiang H. (2014). Integration of rural and urban healthcare insurance schemes in China: An empirical research. BMC Health Serv. Res..

[32] Fu X., Sun N., Xu F., Li J., Tang Q., He J., Wang D., Sun C. (2018). Influencing factors of inequity in health services utilization among the elderly in China. Int. J. Equity Health.

[33] Yu B., Meng Q., Collins C., Tolhurst R., Tang S., Yan F., Bogg L., Liu X. (2010). How does the New Cooperative Medical Scheme influence health service utilization? A study in two provinces in rural China. BMC Health Serv. Res..

[34] Ye C., Duan S., Wu Y., Hu H., Liu X., You H., Wang L., Bogg L., Dong H. (2013). A preliminary analysis of the effect of the new rural cooperative medical scheme on inpatient care at a county hospital. BMC Health Serv. Res..

[35] Wang Y., Jiang Y., Li Y., Wang X., Ma C., Ma S. (2013). Health insurance utilization and its impact: Observations from the middle-aged and elderly in China. PLoS ONE.

[36] Wang L., Wang A., FitzGerald G., Si L., Jiang Q., Ye D. (2016). Who benefited from the New Rural Cooperative Medical System in China? A case study on Anhui Province. BMC Health Serv. Res..

[37] Zhou Z., Gao J., Fox A., Rao K., Xu K., Xu L., Zhang Y. (2011). Measuring the equity of inpatient utilization in Chinese rural areas. BMC Health Serv. Res..

[38] Zhang X., Wu Q., Shao Y., Fu W., Liu G., Coyte P.C. (2015). Socioeconomic inequities in health care utilization in China. Asia Pac. J. Public Health.

[39] Qian Y., Zhou Z., Yan J.e., Gao J., Wang Y., Yang X., Xu Y., Li Y. (2017). An economy-ralated equity analysis of health service utilization by women in economically underdeveloped regions of western China. Int. J. Equity Health.

[40] Zhu D., Guo N., Wang J., Nicholas S., Chen L. (2017). Socioeconomic inequalities of outpatient and inpatient service utilization in China: Personal and regional perspectives. Int. J. Equity Health.

[41] Xiang L., Pan Y., Hou S., Zhang H., Sato K.D., Li Q., Wang J., Tang S. (2016). The impact of the new cooperative medical scheme on financial burden of tuberculosis patients: Evidence from six counties in China. Infect Dis. Poverty.

[42] Zeng Y., Li J., Yuan Z., Fang Y. (2019). The effect of China’s new cooperative medical scheme on health expenditures among the rural elderly. Int. J. Equity Health.

[43] Janković J., Simić S., Marinković J. (2010). Inequalities that hurt: Demographic, socio-economic and health status inequalities in the utilization of health services in Serbia. Eur. J. Public Health.

[44] de Waure C., Bruno S., Furia G., Di Sciullo L., Carovillano S., Specchia M.L., Geraci S., Ricciardi W. (2015). Health inequalities: An analysis of hospitalizations with respect to migrant status, gender and geographical area. BMC Int. Health Hum. Rights.

[45] Lu L., Zeng J., Zeng Z. (2017). What limits the utilization of health services among china labor force? analysis of inequalities in demographic, socio-economic and health status. Int. J. Equity Health.

[46] Wang J., Chen L., Ye T., Zhang Z., Ma J. (2014). Financial protection effects of modification of China’s New Cooperative Medical Scheme on rural households with chronic diseases. BMC Health Serv. Res..

[47] Vancampfort D., Stubbs B., Koyanagi A. (2017). Physical chronic conditions, multimorbidity and sedentary behavior amongst middle-aged and older adults in six low- and middle-income countries. Int. J. Behav. Nutr. Phys. Act..

[48] Tian W.-H., Chen C.-S., Liu T.-C. (2010). The demand for preventive care services and its relationship with inpatient services. Health Policy.

